# Prognostic analysis of interim ^18^F-FDG PET/CT in patients with diffuse large B cell lymphoma after one cycle versus two cycles of chemotherapy

**DOI:** 10.1007/s00259-018-4198-6

**Published:** 2018-10-31

**Authors:** Ling Yuan, Michael C. Kreissl, Liping Su, Zhifang Wu, Marcus Hacker, Jianzhong Liu, Xi Zhang, Yunfeng Bo, Hongyu Zhang, Xiang Li, Sijin Li

**Affiliations:** 1 Department of Nuclear Medicine, First Hospital of Shanxi Medical University, No. 85, Jiefang Road, Taiyuan, 030001 Shanxi China; 2 Department of PET/CT, Tumor Hospital of Shanxi Medical University, Taiyuan, Shanxi China; 3 Department of Radiology and Nuclear Medicine, University Hospital Magdeburg, Magdeburg, Germany; 4 Department of Hematology, Tumor Hospital of Shanxi Medical University, Taiyuan, Shanxi China; 5 Division of Nuclear Medicine, Department of Biomedical Imaging and Image-guided Therapy, Medical University of Vienna, Währinger Gürthel 18-20, Floor 3L, 1090 Vienna, Austria; 6 Department of Pathology, Tumor Hospital of Shanxi Medical University, Taiyuan, Shanxi China

**Keywords:** Diffuse large B cell lymphoma, Interim ^18^F-FDG PET/CT, SUVmax, Deauville five-point scale, MYC gene

## Abstract

**Objectives:**

^18^F-fluorodeoxyglucose (FDG) positron emission tomography (PET) is routinely used in diffuse large B cell lymphoma (DLBCL) for staging, assessment of remission and recurrence, and estimation of therapeutic efficacy. In this study, we aimed to assess the role of an early interim PET/computed tomography (CT) in the evaluation of response in DLBCL.

**Methods:**

Sixty primary DLBCL patients (31 females) were analyzed. Baseline and follow-up ^18^F-FDG PET/CT was performed in patients after one cycle (*n* = 30) and two cycles (n = 30) of chemotherapy. The ΔSUVmax% was calculated. Patients were additionally evaluated using the conventional Deauville five-point scale (D-5PS) system. Fluorescence in situ hybridization (FISH) was employed to characterize the MYC gene status. We determined the optimum cutoff value of ΔSUVmax% using receiver operating characteristic (ROC) analysis. Kaplan–Meier analysis was applied to test for the influence of prognostic values.

**Results:**

The optimal cutoff for the prediction of treatment outcome was a ΔSUVmax% of 57% (after one cycle) and 63% (after two cycles); we could not detect a difference in accuracy with respect to a PET scan performed after one cycle and two cycles of chemotherapy (*P * > 0.05). The ΔSUVmax% and the D-5PS (score 5) showed the highest prognostic value compared to a score of 3 and/or 4 (both after one cycle and two cycles). No significant difference in sensitivity, specificity, accuracy, or the area of under the curve (AUC) of ΔSUVmax% and D-5PS (score 5) was observed between PETs performed after one cycle or two cycles of therapy (*P * > 0.05). ΔSUVmax%, D-5PS (score 5), and MYC gene rearrangement correlated significantly (*P * < 0.001).

**Conclusion:**

Interim ^18^F-FDG PET/CT after one cycle of chemotherapy is feasible and yields similar predictive results as compared to an interim ^18^F-FDG PET/CT after two cycles of chemotherapy in patients suffering from DLBCL. The combination of interim ^18^F-FDG PET/CT with the MYC gene diagnosis might provide increased prognostic value for DLBCL.

## Introduction

Diffuse large B cell lymphoma (DLBCL) is a highly aggressive neoplasm and the most common subtype of non-Hodgkin’s lymphoma (NHL) [1, 2]. Cytotoxic chemotherapy regimens can achieve complete remission. However, it is crucial to identify non-responders and high-risk patients, since lymphomas may be histologically, immunohistochemically, and genetically heterogeneous, making them resistant to chemotherapy [3]. This heterogeneity results in the relapse or progression of 50% of DLBCLs during or after standard treatment [4]. Therefore, it is important to detect patients who will not be sensitive to chemotherapy at an early time point and to guide clinical therapeutic strategies.

Currently, the prognostic assessment of DLBCL is performed according to the International Prognostic Index (IPI) [5, 6]. MYC, BCL2, and BCL6 rearrangements are also essential in providing risk stratification to predict outcome. Nevertheless, an individual IPI cannot accurately identify the specific genetic modification, because of the heterogeneity of these neoplasms [7, 8].

Interim ^18^F-FDG PET has shown promising results in the prognostic evaluation of patients after treatment [9–12]. Lymphoma manifestations which have a decreased glucose metabolism on interim staging are more likely to respond to chemotherapy [13, 14]. Most investigations have focused on the use of interim PET after two cycles of chemotherapy to predict therapeutic outcome. Even though the results of those trials were very promising, there is, however, still a controversy about the utility of the interim PET to change treatment, its role in clinical trials, or whether to analyze this PET with Deauville five-point scale(D-5PS) or with ∆SUV [15, 16]. In spite of this, there is an increasing clinical interest in the use of interim ^18^F-FDG PET/CT, particularly after one cycle of chemotherapy, to predict therapeutic response and outcome.

The maximum standardized uptake value reduction proportion (ΔSUVmax%) and the D-5PS are usually applied to assess therapeutic effects during/after chemotherapy, but the criteria still remain controversial [14, 17–19]. The prognostic value of ΔSUVmax% and D-5PS derived from interim PET evaluation (after one cycle compared to two cycles of chemotherapy) has not been clarified for DLBCL.

Rearrangement of MYC is known to be associated with poor prognosis in DLBCL patients. This rearrangement is found in 5–15% of DLBCL cases and is connected with resistance to chemotherapy, disease progression, and poor prognosis in late-stage disease [20–24]. Therefore, we also assessed the MYC status together with the parameters obtained by FDG PET as a prognostic marker.

In this present study, we sought to evaluate the prognostic value of ΔSUVmax% derived from interim FDG PET/CT after one cycle as compared to two cycles of chemotherapy in DLBCL patients. We also compared the two time points using the conventional D-5PS evaluation and determined the best cutoff of the D-5PS to predict the progression free-survival (PFS). The impact of MYC rearrangement was also assessed as a secondary objective.

## Materials and methods

### Patients and treatment

A retrospective analysis was performed on a cohort of 60 DLBCL patients (29 male and 31 female, age: 51 ± 16.2 yrs. with range: 18–81 yrs.) who were prospectively enrolled in a larger trail. All patients had a DLBCL confirmed by pathology as the only type of malignancy. Seventeen patients underwent CHOP (cytoxan, adriamycin, vincristine, and prednisone) and 43 patients underwent R-CHOP therapy (rituximab) in cycles of 21 days each. Informed consent was obtained according to the protocol approved by the local ethics committee.

### International prognostic index (IPI)

The IPI score included six risk factors: age > 60 years (+); age > 70 years (−); ECOG PS >1 (+); elevated serum lactate dehydrogenase (LDH; +); extranodal sites >1 (+); and stage III or IV (+). The number of risk factors determined the category of risk, as follows: low risk (score 0–1); low intermediate risk (score 2); high intermediate risk (score 3); and high risk (score 4–5).

### ^18^F-FDG pet/CT

^18^F-FDG PET/CT post-chemotherapy examinations were scheduled 1 week before the next course of chemotherapy. The patients who underwent ^18^F-FDG PET/CT after one cycle (*n* = 30) and two cycles (n = 30) of chemotherapy were randomly assigned to these two groups. A hybrid PET/CT scanner (GE Discovery STE, USA) was used for staging and restaging of lymphoma. Image data was acquired 60 min after the injection of 4.44–5.55 MBq ^18^F-FDG per kg body weight. The subsequent whole-body ^18^F-FDG PET (from the head to the middle part of the thigh) was performed in three-dimensional acquisition mode with 6–8 bed positions and 2.5 min/position using the CT data for PET image attenuation correction. The following parameters for the CT scan were used: tube voltage of 120 kV; current of 180 mA; pitch of 0.938:1; slice thickness of 3.75 mm; and a single-round tube rotation time of 0.8 s. The PET images were reconstructed by ordered-subsets expectation maximization (OSEM), 2 iterations and 20 subsets and a matrix size of 128 × 128 pixels were used in the reconstruction.

### Image analysis

Visual and semi-quantitative analysis was employed. Two board-certified nuclear medicine physicians (Yuan L. and Wu Z. F.) experienced in reading PET/CT evaluated the PET data. For semi-quantitative analysis, the highest ^18^F-FDG uptake was determined based on an region of interest (ROI) method. The SUVmax and the relative decrease of the SUVmax (ΔSUVmax%) between pre-chemotherapy (preSUVmax) and post-chemotherapy (postSUVmax) were calculated [ΔSUVmax% = (preSUVmax – postSUVmax)/preSUVmax × 100%].

In addition, the standard D-5PS scoring system was used to assess treatment response qualitatively as follows: score 1: no residual disease; score 2: if the residual tumor uptake of ^18^F-FDG was less than or equal to the mediastinal blood pool; score 3: if the standard of the mediastinal blood pool was less than the residual lesioned ^18^F-FDG uptake, and less than or equal to the liver uptake; score 4: if the ^18^F-FDG uptake of residual lesions was slightly greater than that of the liver; and score 5: if the ^18^F-FDG uptake in residual lesions was significantly more than that in the liver (over two times) or new lesions were detected.

### Immunohistochemical staining

CD20, BCL-2 (Gene Tech Biotechnology, Shanghai, China), CDl0 (Golden Bridge Biotechnology, Beijing, China), BCL-6, and MUM1 (Maxin Biotechnology,Fuzhou, China) were labeled via a two-step process using EnVision. A positive staining was defined as the appearance of distinct positions of brown-yellow or brown particles. The immunochemical positive reactivity patterns of CD20, CDl0, and BCL-2 were identified in the membrane and those of BCL-6 and MUM1 were identified in the nucleus. According to the expression levels of CDl0, BCL-6, and MUM1, DLBCL was classified as germinal center B cell (GCB) or non-GCB [25].

### Fluorescence in situ hybridization (FISH) testing

An MYC dichroic separation rearrangement probe (SPEC LSl MYC Dual Color Breakapart Probe, ZytoLight, IL, USA) was used. Normal mitosis interphase nuclei could be seen as two fused signals (Fig. 3A). MYC gene rearrangement was deemed to be positive if over 3.1% of the tumor cell nuclei appeared as monochromatic signals. (Fig. 3B).

### Clinical and follow-up assessment

Associations between tumor stage and patients’ baseline characteristics were assessed after one or two cycles of chemotherapy separately, including gender, age, Ann Arbor stage, IPI score, immunophenotyping, treatment regimen, hepatitis B virus infection (HBV), LDH level, presence of B symptoms, bone marrow infiltration, imaging data (CT, ultrasound, and PET/CT), immunohistochemical results (CD20, CDl0, BCL-2, BCL-6, and MUM1), and MYC gene rearrangement. The range of follow-up time was from 6 to 71 months. The primary end-point of the study was PFS, which was defined as the time from diagnosis to the first occurrence of progression, relapse, death due to any cause, or last follow-up.

### Statistics

Measured data within a normal distribution are expressed as the mean ± standard deviation (SD). Based on the D-5PS system, we compared the prognostic value of the criteria for the D-5PS score 3 (1–2 considered negative), score 4 (1–3 considered negative), and score 5 (1–4 considered negative) in terms of the sensitivity, specificity, accuracy, and area under the curve (AUC). Receiver operating characteristic (ROC) analysis was used to determine the optimal cutoff value for ΔSUVmax% in predicting disease progression or death. Sensitivity, specificity, accuracy, and AUC of prognosis for an optimal cutoff ΔSUVmax% were also compared between two cycles. Spearman’s rank correlation coefficient (Spearman’s rho) between D-5PS (score 5), MYC gene rearrangement, and ΔSUVmax% were calculated. Univariate linear regression analysis was performed for estimating the correlation between clinical variables and the cutoff value of ΔSUVmax% or D-5PS after one or two cycles of chemotherapy. Chi square (χ^2^) tests were used to compare categorical variables. Survival rate analysis was implemented by the Kaplan–Meier and log-rank test. Spearman correlation was used to evaluate the relationships between ΔSUVmax%, D-5PS (score 5), and the presence of MYC gene rearrangement. Differences between the results in comparative tests were considered significant if the two-sided *P* value was <0.05. All statistical analyses were performed with SPSS 22.0.

## Results

### Clinical characteristics and protein expression characteristics

The clinical characteristics of all DLBCL, including staging, IPI scores, and chemotherapy regimens, can be found in Table 1. In terms of outcome, 15 patients showed progression (*n* = 9 post 1 cycle, *n* = 6 post 2 cycles), 42 patients reached complete response (*n* = 19 post 1 cycle, *n* = 23 post 2 cycles), and three patients' response (n = 2 post 1 cycle, n = 1 post 2 cycles) showed partial remission during the follow-up period. The association between the prognosis (PFS) of DLBCL patients and age, gender, B symptoms, HBV or bone marrow involvement, Ann Arbor stage, treatment regimen, IPI score, immunophenotypes of GCB, and serum LDH level after one and two cycles of chemotherapy were separately listed in Table 1. The prognosis (PFS) of patients was significantly related to the IPI score and MYC gene rearrangement after each cycle of chemotherapy (*P* < 0.05; Table 1). Patients’ clinical data mainly including Ann Arbor Stage, IPI, and MYC gene rearrangement significantly correlated with ΔSUVmax% cutoff value and D-5PS (score 5). Detailed linear correlation regression analysis results are listed in Table 2.

**Table 1:** Patients’ clinical characteristics and association with tumor progression

Clinical characteristics	One cycle			Two cycles		
	Progress rate	χ^2^	*P* value	Progress rate	χ^2^	*P* value
Gender						
Male	37.5% (6/16)	0.92	0.338	0% (0/13)	5.74	0.017
Female	21.4% (3/14)			35.3% (6/17)		
Age						
<60	17.6% (3/17)	2.85	0.091	10.5% (2/19)	2.91	0.088
≥60	46.2% (6/13)			36.4% (4/11)		
Ann Arbor Stage						
I-II	14.3% (2/14)	3.09	0.079	0% (0/11)	4.34	0.037
III-IV	43.8% (7/16)			31.6% (6/19)		
IPI						
0–2	15.8% (3/19)	4.98	0.026	6.3% (1/16)	4.05	0.044
3–5	54.5% (6/11)			35.7% (5/14)		
Immunophenotype						
GCB	0% (0/10)	6.43	0.011	15.4% (2/13)	0.31	0.580
Non-GCB	45.0% (9/20)			23.5% (4/17)		
Treatment regimen						
R-CHOP	21.1% (4/19)	1.98	0.160	16.7% (4/24)	0.83	0.361
CHOP	45.5% (5/11)			33.3% (2/6)		
HBV						
–	28.6% (8/28)	0.41	0.523	14.8% (4/27)	4.54	0.033
+	50.0% (1/2)			66.7% (2/3)		
LDH						
<248 U/L	17.6% (3/17)	2.85	0.091	11.8% (2/17)	1.66	0.197
≥248 U/L	46.2% (6/13)			30.8% (4/13)		
B symptoms						
Yes	33.3% (2/6)	0.04	0.842	0% (0/4)	1.15	0.283
No	29.2% (7/24)			23.1% (6/26)		
Bone marrow infiltration						
Yes	66.7% (2/3)	2.13	0.144	40.0% (2/5)	1.50	0.221
No	25.9% (7/27)			16.0% (4/25)		
MYC gene rearrangement						
–	0% (0/18)	19.29	<0.001	0% (0/24)	30.00	<0.001
+	75.0% (9/12)			100% (6/6)		
CD10 protein						
–	40.9% (9/22)	4.68	0.031	22.7% (5/22)	0.38	0.536
+	0% (0/8)			12.5% (1/8)		
MUM1 protein						
–	22.2% (2/9)	0.37	0.543	22.2% (2/9)	0.04	0.842
+	33.3% (7/21)			19.0% (4/21)		
BCL-6 protein						
–	30.0% (3/10)	0	1.000	18.2% (2/11)	0.04	0.850
+	30.0% (6/20)			21.1% (4/19)		
BCL-2 protein						
–	10.0% (2/20)	11.43	0.001	16.0% (4/25)	1.50	0.221
+	70.0% (7/10)			40.0% (2/5)		

IPI: International Prognostic Index; GCB: germinal center B-cell; CHOP: cyclophosphamide, doxorubicin, vincristine, and prednisone; HBV: hepatitis B virus infection; LDH: serum lactate dehydrogenase. A chi-square test was used to test the significance of the association between tumor status and patients’ baseline characters

**Table 2:** Correlation between ΔSUVmax%/D-5PS (score 5) with clinical characteristics

	ΔSUVmax%				D-5PS (score 5)			
	One cycle		Two cycles		One cycle		Two cycles	
	Coefficients	*P* value	Coefficients	*P* value	Coefficients	*P *value	Coefficients	*P *value
Gender	0.24	0.205	−0.31	0.100	−0.24	0.201	0.23	0.227
Age	−0.28	0.131	−0.10	0.592	0.28	0.128	0.15	0.425
Ann Arbor Stage	−0.35	0.060	−0.47	0.009	0.20	0.295	0.62	<0.001
IPI	−0.54	0.002	−0.45	0.012	0.41	0.025	0.63	<0.001
MYC gene	−0.073	<0.001	−0.69	<0.001	0.65	<0.001	0.64	<0.001
Treatment regimen	0.34	0.070	0.32	0.087	−0.41	0.025	−0.24	0.199
Immunophenotype	−0.39	0.036	−0.04	0.846	0.37	0.042	0.29	0.128
B symptoms	−0.28	0.128	0.01	0.950	0.22	0.247	0.16	0.394
Bone marrow infiltration	−0.26	0.172	−0.05	0.795	0.27	0.154	0.24	0.199
LDH	−0.47	0.009	−0.19	0.313	0.37	0.044	0.40	0.027
HBV	−0.26	0.165	−0.21	0.256	0.20	0.295	0.26	0.173
CD10	0.30	0.112	0.18	0.354	−0.22	0.236	−0.27	0.156
MUM1	−0.12	0.513	−0.01	0.959	0.23	0.220	0.24	0.198
BCL-6	−0.17	0.379	−0.32	0.088	0.06	0.754	−0.03	0.861
BCL-2	−0.61	<0.001	−0.22	0.239	0.62	<0.001	0.34	0.069

### Comparative analysis

**Fig. 1:**
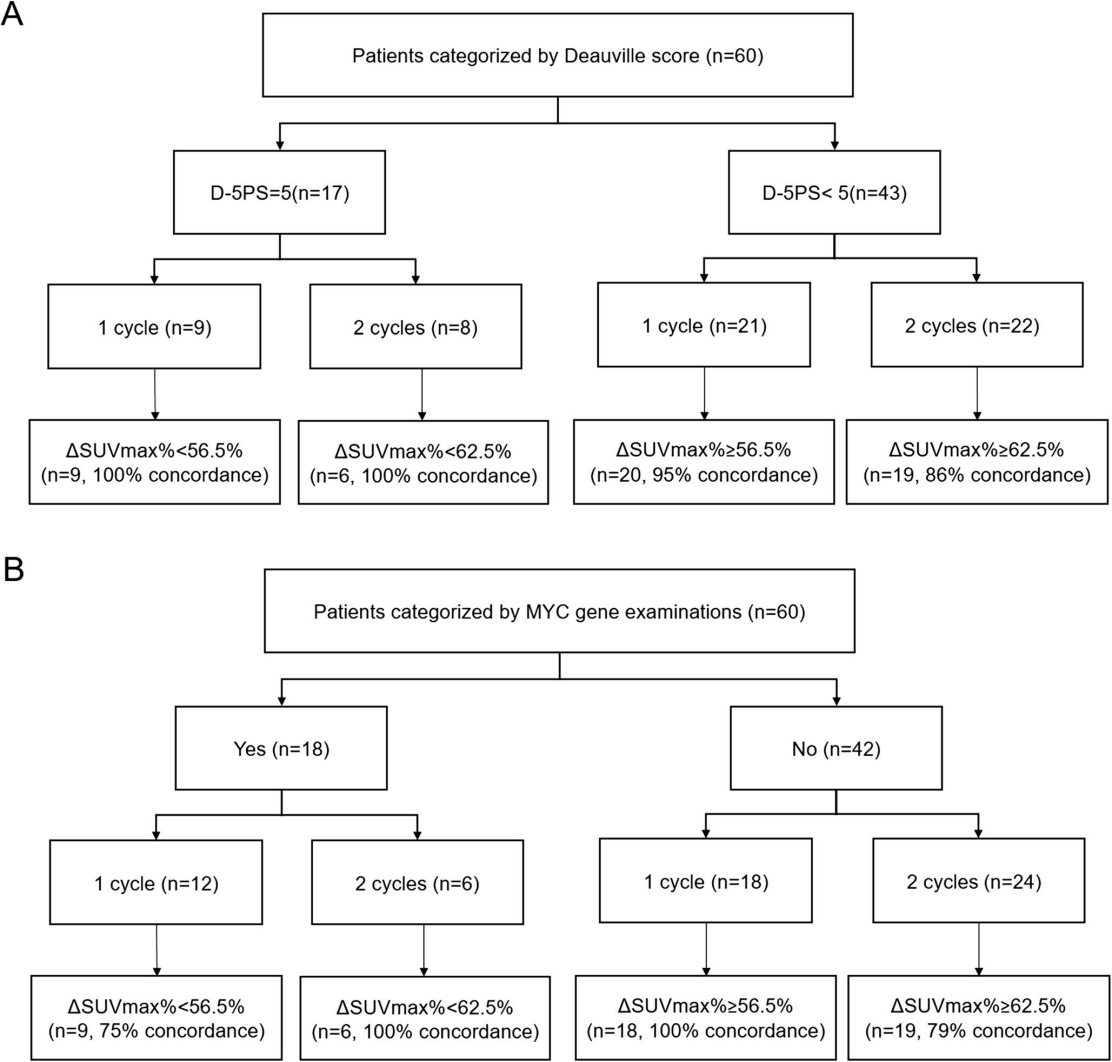
Patients categorized by the ΔSUVmax% method with MYC gene examinations and Deauville sore methods. All sub-grouped patients were also grouped by the cutoff ΔSUVmax% value of 57% after 1 cycle of therapy and a cutoff ΔSUVmax% value of 63% after 2 cycles of therapy. Concordance indicated the percentage of patients categorized by different criteria after one or two cycles of chemotherapy

Based on ROC assessment, the D-5PS (score 5) showed the highest prognostic values for disease progression status compared to the D-5PS (score 4) and D-5PS (score 3). The data on the sensitivity, specificity, accuracy, and AUC of D-5PS (score 3), D-5PS (score 4), and D-5PS (score 5) are listed in Table 3. Moreover, the best cutoff for ΔSUVmax% as a prognostic parameter for disease progression after one cycle of chemotherapy was found to be 57%; after two cycles it was 63%. We did not detect a significant difference between the prognostic values (sensitivity, specificity, and the AUC) obtained between after one cycle as compared to two cycles when applying the ΔSUVmax% cutoff or the D-5PS (score 5) method (Table 4). There also was no significant difference observed between the ΔSUVmax% and D-5PS (score 5) at either time point (Table 5). The flowchart of patients categorized by D-5PS (score 5) and MYC gene status is displayed in Fig. 1. There was a good concordance for the prognosis of disease between the ΔSUVmax% method, MYC gene examinations, and Deauville score methods.

**Table 3:** Comparison of sensitivity, specificity, and accuracy of D-5PS (score 3), D-5PS (score 4), and D-5PS (score 5) methods for the prediction of progressive disease after one cycle and two cycles of therapy

D-5PS	One cycle			Two cycles		
	Score 3	Score 4	Score 5	Score 3	Score 4	Score 5
Sensitivity	38.1% (8/21)	44.4% (8/18)	88.9% (8/9)*	23.1% (6/26)	27.3% (6/22)	62.5% (5/8)*
Specificity	88.9% (8/9)	91.7% (11/12)	95.2% (20/21)	100% (4/4)	100% (8/8)	95.5% (21/22)
Accuracy	53.3% (16/30)	63.3% (19/30)	93.3% (28/30)*	33.3% (10/30)	46.7% (14/30)	86.7% (26/30)*
AUC (95% CI)	0.635 (0.427–0.843)	0.706 (0.513–0.900)	0.921 (0.000–1.000)	0.583 (0.347–0.820)	0.667 (0.458–0.875)	0.854 (0.000–1.000)

D-5PS (score 3 scores): D-5PS with score 1–2 considered negative

D-5PS (score 4 scores): D-5PS with score 1–3 considered negative

D-5PS (score 5 scores): D-5PS with score 1–4 considered negative

*AUC*: area under the receiver operating characteristic curve; *CI* confidence interval

*: *P* < 0.05 considered statistically significant

**Table 4:** Comparison of sensitivity, specificity, and accuracy of D-5PS (five scores) and ΔSUVmax% with cutoff value for disease prognosis between time points after one and two cycles of therapy

	ΔSUVmax%				D-5PS (score 5)			
	One cycle	Two cycles	χ^2^	*P* value	One cycle	Two cycles	χ^2^	*P* value
Sensitivity	80% (8/10)	54.5% (6/11)	1.53	0.228	88.9% (8/9)	63% (5/8)	1.64	0.108
Specificity	95% (19/20)	100% (19/19)	0.98	0.349	95.2% (20/21)	95.5% (21/22)	0.001	1.000
Accuracy	90% (27/30)	83.3% (25/30)	0.58	0.464	93.3% (28/30)	86.7% (26/30)	0.74	0.416
AUC (95% CI)	0.897 (0.000–1.000)	0.896 (0.773–1.000)			0.921 (0.000–1.000)	0.854 (0.000–1.000)		

For positive disease progression: cutoff value of ΔSUVmax% after one cycle was 57% and the cutoff value of ΔSUVmax% after two cycles was 63%

D-5PS (score 5): 5PS with score 1–4 considered negative

*AUC*: area under the ROC curve; *CI* confidence interval

**Table 5:** Comparison of sensitivity, specificity, and accuracy for disease prognosis between D-5PS (five points) and ΔSUVmax% at time points after one and two cycles

	One cycle				Two cycles			
	ΔSUVmax%	D-5PS (score 5)	χ^2^	*P* value	ΔSUVmax%	D-5PS (score 5)	χ^2^	*P* value
Sensitivity	80% (8/10)	88.9% (8/9)	0.21	0.596	54.5% (6/11)	62.5% (5/8)	0.12	0.736
Specificity	95% (19/20)	95.2% (20/21)	0.001	1.000	100% (19/19)	95.5% (21/22)	0.89	0.375
Accuracy	90% (27/30)	93.3% (28/30)	0.22	0.640	83.3% (25/30)	86.7% (26/30)	0.13	0.728
AUC (95% CI)	0.897 (0.000–1.000)	0.921 (0.000–1.000)			0.896 (0.773–1.000)	0.854 (0.000–1.000)		

For positive disease progression: cutoff value of ΔSUVmax% after one cycle was 57% and the cutoff value of ΔSUVmax% after two cycles was 63%

D-5PS (score 5): 5PS with score 1–4 considered negative

*AUC*: area under the ROC curve; *CI* confidence interval

### Survival analysis for ΔSUVmax%, D-5PS, and MYC gene rearrangement

The prognostic results using a cutoff for ΔSUVmax% were significantly correlated with the MYC gene rearrangement and the D-5PS (score 5), both after cycle 1 and cycle 2 of chemotherapy (*P* < 0.001; Table 6).

**Table 6:** Comparison of Spearman’s rho of D-5PS (score 5), MYC gene rearrangement, and ΔSUVmax% with a cutoff value for the prediction of progressive disease after one cycle and two cycles of therapy

Correlation	One cycle		Two cycles	
	r	*P* value	r	*P* value
ΔSUVmax%/MYC	0.72	<0.001	0.66	<0.001
ΔSUVmax%/D-5PS	0.93	<0.001	0.79	<0.001
D-5PS*/*MYC	0.65	<0.001	0.64	<0.001

Prediction of progressive disease: ΔSUVmax% < 57% after one cycle and ΔSUVmax% < 63% after two cycles; D-5PS (score 5) considered positive, MYC: positive MYC rearrangement

**Fig. 2:**
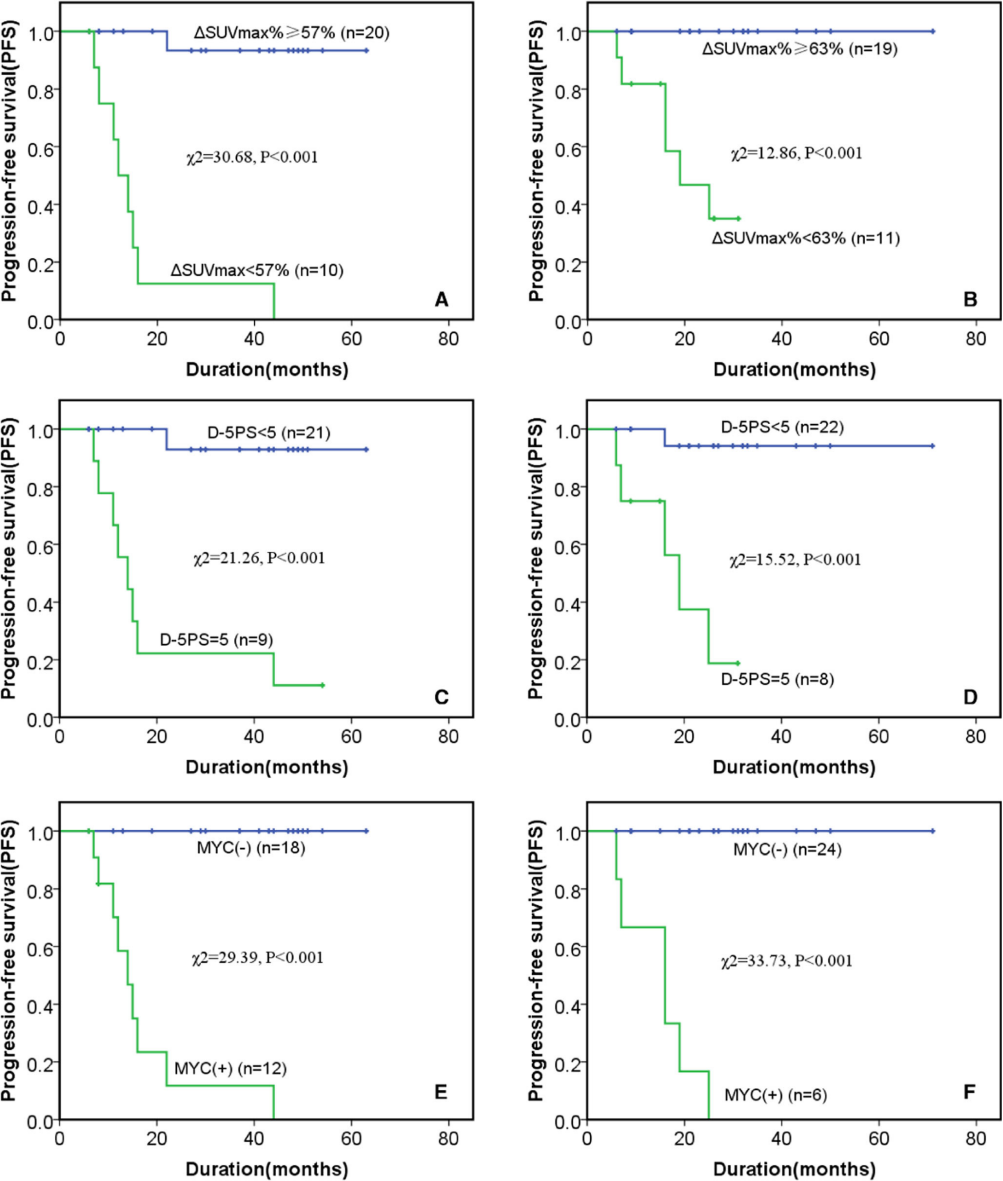
Progression-free survival of categorized patients according to ΔSUVmax% cutoff value (A and B), D-5PS (score 5; C and D), and MYC gene status (E and F). A, C, and E refer to patients after one cycle of chemotherapy; B, E, and F refer to patients after two cycles of chemotherapy

Patients were categorized according to the cutoff value of ΔSUVmax% (57%) at one cycle of chemotherapy and ΔSUVmax% (63%) after two cycles of chemotherapy. The PFS was analyzed by Kaplan–Meier survival analysis (Fig. 2).

**Fig. 3:**
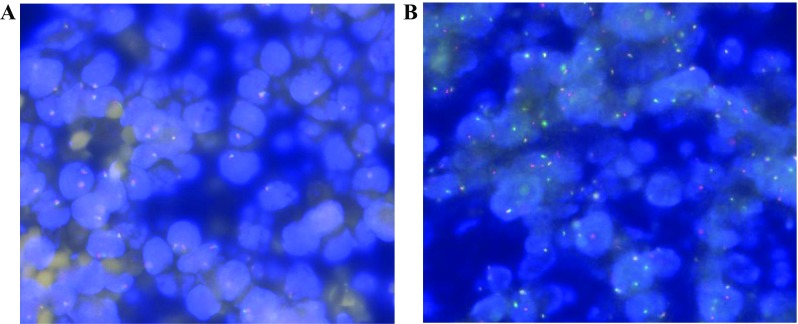
Representative photomicrographs of neoplastic cells for MYC gene rearrangement examinations. A. Negative MYC gene rearrangement: mitosis interphase nuclei can be seen as two fused signals (red and green); B, positive MYC gene rearrangement: mitosis interphase nuclei can be seen as two separated signals

We found a significant difference in PFS in both groups, when the specific cutoff for ΔSUVmax was applied, with the group imaged after 1 cycle at 57% (χ^2^ = 30.68, *P* < 0.001; Fig. 2A) and the group imaged after 2 cycles at 63% (χ^2^ = 12.86, *P *< 0.001; Fig. 2B). Patients were also categorized according to the D-5PS scoring method (score < 5 or score = 5) at both time points, and the PFS also significantly differed between these categorized groups after one cycle (χ^2^ = 21.26, *P *< 0.001; Fig. 2C) and after two cycles (χ^2^ = 15.52, *P *< 0.001; Fig. 2D). Patients with an MYC gene rearrangement had a significantly worse PFS than patients without an MYC gene rearrangement at after one cycle (χ^2^ = 29.39, *P *< 0.001; Fig. 2E) and after two cycles (χ^2^ = 33.73, *P *< 0.001; Fig. 2F). Representative photomicrographs of neoplastic cells for MYC gene rearrangement exanimations are shown in Fig. 3.

In the cohort of patients with an MYC rearrangement (*n* = 18), when applying the ΔSUVmax% cutoff value, we also found that three false-positive patients had no progression, with values of ΔSUVmax% and D-5PS of 71.7/D-5PS (score 4), 86.1%/D-5PS (score 4), and 46.1%/D-5PS (score 4) each after 1 cycle.

## Discussion

^18^F-FDG PET/CT has been widely used to guide therapy for DLBCL [26, 27]. However, the prognostic value of the current interpretation standard of the ΔSUVmax% and the D-5PS scale in the clinical practice still remains controversial [18, 28–32]. In this study, we compared the prognostic value of early PET scans already after one cycle compared to two cycles of chemotherapy using the ΔSUVmax% and D-5PS methods. We conducted an ROC curve analysis for the ΔSUVmax% of DLBCL during the early stage of chemotherapy to identify the best threshold value for predicting tumor progression. The best cutoff values of ΔSUVmax% for disease prognostication were 57 and 63% for one cycle and two cycles of chemotherapy, respectively, and there was no significant difference between the time points.

In a pioneering interim ^18^F-FDG PET/CT study for DLBCL, Fuertes et al. [28] showed a promising prognostic value using the criteria of ΔSUVmax% > 75% following 2 or 3 cycles of chemotherapy. Casasnovas et al. [18] performed an interim ^18^F-FDG PET/CT after two cycles of chemotherapy, and demonstrated that ΔSUVmax% has a high prognostic value in DLBCL and the optimized cutoff value of ΔSUVmax% was 66% (after 2 cycles) and 70% (after 4 cycles). This optimal cutoff after 2 cycles was close to the cutoff point of 66% reported by Casasnovas et al. [18]. Interestingly, we found that ΔSUVmax% after one cycle of treatment was only slightly inferior in terms of specificity as compared to ΔSUVmax% after two cycles (95% vs, 100%); in contrast, both sensitivity (80% vs. 54.5%) and accuracy (90% vs 83.3%) tended to be higher.

The D-5PS system is also commonly used to evaluate interim PET/CT scans. Published studies showed a high prognostic value of D-5PS (score 4) for interim ^18^F-FDG PET/CT scans in patients with DLBCL. Nevertheless, Casasnovas et al. [18] and Itti et al. [29] demonstrated a poor prognostic value for the D-5PS (score 4) in interim ^18^F-FDG PET scans for the prediction of PFS after either two or four cycles of chemotherapy. Mylam et al. [30] demonstrated the limited prognostic value of D-5PS (score 4) in a study of 112 DLBCL cases after 1 cycle of chemotherapy, but the D-5PS (score 5) showed a relatively higher prognostic value. Kim et al. [31] also showed that PFS was inferior in patients with a score of 5 on an interim PET scan compared to those with a score between 1 and 4. In this present study, D-5PS (score 5) showed the highest outcome prediction ability compared to either D-5PS (score 3) or D-5PS (score 4), which is in keeping with the study by Mylam et al. [30] and Kim et al. [31]. However, the false-positive ratio was higher during the early course of chemotherapy. The reason for these results might be found in the heterogeneity of tumors, which could have lead to the disparate therapeutic responses. Also, in 2006, Kostakoglu et al. published an article assessing the value of an interim PET after one cycle of therapy. Unfortunately, the results for the visual assessment, which were very good with a sensitivity of 100% and specificity of 94%, cannot be compared to our study because another classification system was used. In terms of quantitative assessment, the colleagues found the best cutoff to be an SUVmax of 1.75 (sensitivity 92%; specificity 84%). Also, this value cannot be compared to our study because in their study, older and various types of PET scanners were used. However, what this study shows is, that in their study, as well as in ours, the interim PET has the potential for an excellent discrimination between responding and non-responding patients.

It has been previously demonstrated that the combination of molecular and imaging characteristics at diagnosis could lead to a more accurate selection of patients, to increase therapy tailoring [33], so we also included the assessment of MYC gene rearrangement into our analysis. In our present study, an MYC gene rearrangement by FISH was found in the 30% of the patient population, which is significantly higher as compared to 5–15% reported in previous studies [20–23]. This may be related to the higher proportion of patients in the high-risk group classified as stage IV and the IPI scores. Our study showed that the prognostic value of ΔSUVmax% was positively correlated with an MYC gene rearrangement and the D-5PS (score 5) either after one cycle or two cycles. Even though an MYC gene rearrangement was present, 3 out of 18 patients had no disease progression. Two were classified correctly as responders with a ΔSUVmax% of 71.66% and a D-5PS (score 4), a ΔSUVmax% of 86.09%/D-5PS (score 4); one was false negative with a ΔSUVmax% under the threshold (46.14%), but at the same time, a D-5PS (score 4) indicating response according to our established threshold. Our data, with the limitations mentioned below, is very much in keeping with previously published data on the influence of the molecular characteristics and PET-derived parameters on overall and progression free survival [33]. Here, the PET-derived initial total tumor metabolic volume was found to be a good predictor of the further course.

### Limitations

This unicentric retrospective analysis has several limitations. Due to the limited number of patients, a multi-factor synergistic regression analysis for ΔSUVmax%, D-5PS (score 5), and MYC gene status was not possible. Thus, the joint prognostic value with multiple factors for PFS could not be compared at one and two cycles. In addition, heterogeneity was introduced into the trial because two different systemic treatment regimens were used. In our patient population, of the 43 patients who received R-CHOP therapy, 8 patients progressed (18.6%), and of the 17 patients who received CHOP therapy, 7 patients showed progressive disease (41.18%). However, using a chi-square or exact test addressing the type of systemic treatment, *P* values proved to be not significant (*P* = 0.069 or *P* = 0.099, respectively); therefore, the use of two chemotherapy regimes is most likely not resulting in a significant effect in terms interpretability of the results.

## Conclusion

An interim ^18^F-FDG PET/CT examination performed already after one cycle of chemotherapy was found to have equal power for the prediction of prognosis in DLBCL patients as compared to interim staging after two cycles. Therefore, an earlier prediction of response seems to be feasible, but should be confirmed in the context of a larger clinical trial.
